# Magnesium Is a Vital Ion in the Body—It Is Time to Consider Its Supplementation on a Routine Basis

**DOI:** 10.3390/clinpract14020040

**Published:** 2024-03-22

**Authors:** Ákos Géza Pethő, Tibor Fülöp, Petronella Orosz, Mihály Tapolyai

**Affiliations:** 1 Department of Internal Medicine and Oncology, Faculty of Medicine, Semmelweis University, 1085 Budapest, Hungary; 2 Medicine Service, Ralph H. Johnson VA Medical Center, Charleston, SC 29401, USA; fulopt@musc.edu (T.F.); dr.tapolyai.mihaly@sztmargit.hu (M.T.); 3 Department of Medicine, Division of Nephrology, Medical University of South Carolina, Charleston, SC 29425, USA; 4 Bethesda Children’s Hospital, 1146 Budapest, Hungary; orosz.petronella@bethesda.hu; 5 Department of Pediatrics, Faculty of Medicine, University of Debrecen, 4032 Debrecen, Hungary; 6 Department of Nephrology, Szent Margit Kórhaz, 1032 Budapest, Hungary

**Keywords:** bone disease, cardiovascular disease, chronic diseases, diabetes, COVID-19, inflammation, magnesium, mineral metabolism, renal disease

## Abstract

The importance of maintaining proper magnesium intake and total body magnesium content in preserving human health remains underappreciated among medical professionals and laymen. This review aimed to show the importance of hypomagnesemia as a modifiable risk factor for developing disease processes. We searched the PubMed database and Google Scholar using the keywords ‘magnesium’, ‘diabetes’, ‘cardiovascular disease’, ‘respiratory disease’, ‘immune system’, ‘inflammation’, ‘autoimmune disease’, ‘neurology’, ‘psychiatry’, ‘cognitive function’, ‘cancer’, and ‘vascular calcification’. In multiple contexts of the search terms, all reviews, animal experiments, and human observational data indicated that magnesium deficiency can lead to or contribute to developing many disease states. The conclusions of several in-depth reviews support our working hypothesis that magnesium and its supplementation are often undervalued and underutilized. Although much research has confirmed the importance of proper magnesium supply and tissue levels, simple and inexpensive magnesium supplementation has not yet been sufficiently recognized or promoted.

## 1. Introduction

Magnesium is a divalent cation found abundantly inside cells. Many studies have discussed Mg homeostasis in detail [1,2,3]. However, we will only focus on the important physiological processes that can be influenced by simple and inexpensive oral magnesium supplementation techniques. Magnesium is crucial for maintaining normal cellular physiology and metabolism as it acts as a cofactor for multiple enzymes, regulating ion channels and energy generation [1]. Magnesium plays a vital role in various aspects of vascular function, including vascular tone, the development of atherosclerosis and blood clot formation, the calcification of blood vessels, and the growth and movement of cells in the inner lining and smooth muscle of blood vessels. Moreover, magnesium plays a vital role in various biological processes, including signal transduction, oxidative phosphorylation, glycolysis, and the production of proteins and DNA. It should be mentioned that the human body contains 25 g or 1000 mmol of magnesium, 98% of which is distributed in soft tissues (38%) and bones (60%) [3,4]. The serum fraction constitutes approximately 1% of the total and is biologically active, owing to its ionization [1,5]. Many factors can lead to Mg deficiency in the body [6]. Table 1 summarizes the most critical processes that ensure magnesium homeostasis. Mg deficiency is most often caused by insufficient intake or increased excretion. It can develop very often as a side effect of many medications [7].

According to some authors, at least 30% of the adult population does not consume the daily magnesium requirement through their diet [8]. 

Insufficient magnesium intake in one’s diet has been found to increase the probability of mortality and the development of several health conditions, including cardiovascular diseases, diabetes, stroke, cancer, and bone fractures [9]. Studies have shown that low magnesium levels in elderly patients can have negative impacts on bone health, insulin sensitivity, glycosylation, and heart and blood vessel function. Therefore, it is essential to be aware of these potential risks. Furthermore, it may have a detrimental effect on cognitive function and mood. Therefore, immediate action to prevent hypomagnesemia is crucial to maintain a healthy aging process in elderly patients [10]. 

However, hypermagnesemia is not a common serum electrolyte abnormality. The largest contributing risk factor is reduced renal function, which is observed in approximately 10 percent of hospitalized patients. It is essential to monitor hypermagnesemia patients on magnesium supplementation when encountering acute illness and renal failure together, as their condition may require immediate medical intervention [11]. Symptoms of high blood magnesium levels include weakness, nausea, dizziness, and confusion, which typically occur at levels lower than 7.0 mg/dL. As magnesium levels increase (7–12 mg/dL), severe neurological symptoms may appear, including decreased reflexes, worsened confusion, drowsiness, bladder paralysis, flushing, headache, and constipation. Sometimes, blurred vision may also occur. In cases where magnesium levels exceed 12.0 mg/dL, the individual may experience more severe symptoms such as muscle paralysis, paralytic ileus, decreased breathing rate, low blood pressure, and changes in electrocardiogram (ECG) readings. If magnesium levels exceed 15.0 mg/dL, it can result in coma and immediate cardiac arrest [12,13].

In our review, we aimed to carefully analyze the importance of hypomagnesemia as a modifiable risk factor using the information provided in the literature. Our hypothesis was that hypomagnesemia plays an essential role in the generation and acceleration of multiple diseases. It is crucial to emphasize that substituting this essential ion can effectively ameliorate certain diseases; doing so can lead to cost-effectiveness in various areas, making it a practical and advantageous preventive intervention. The prevention of diabetes and cancer has significant implications for healthcare systems. A reduction of up to 30% in the incidence of these ailments constitutes a substantial achievement in the field of public health. Such accomplishments could have a profound and positive impact on the healthcare system by reducing the burden of care and treatment.

## 2. Search Methods

We conducted a comprehensive search of medical databases, including PubMed and Google Scholar, using specific keywords such as magnesium, diabetes, cardiovascular disease, respiratory disease, immune system, inflammation, autoimmune disease, neurology, psychiatry, cognitive function, cancer, and ‘vascular calcification’. The purpose of this search was to identify the relevant scientific literature on these topics. The search process was performed systematically and the identified articles were carefully evaluated and reviewed for their relevance and quality. We followed the guidelines for literature searches and manuscript preparation [14,15]. The present study established inclusion criteria for all articles published by 20 August 2023, including systematic reviews and studies involving humans or animals. The search was not restricted to a specific start time; instead, the first publication available for the given keyword was used as the baseline. To avoid duplication, all the repeated publications were excluded from the search. Relevant publications were identified via a two-pronged approach, including a backward search that involved examining the references of retrieved articles and an ahead search that involved scrutinizing more recent publications that cited the retrieved papers.

Our hypotheses were extracted from studies that addressed the review objectives. The main focus of data collection for each study was to identify possible connections to our theory. To ensure accuracy, three authors independently selected each retrieved article for eligibility and extracted the study data. Any discrepancies between the first and senior authors (GP, MT, and TF) were resolved through discussion and agreement.

## 3. Results

Our narrative review statement aims to draw attention to reducing easily addressable risk factors such as hypomagnesemia in the development of many diseases. We were interested in assessing the number of publications that appeared using the search terms and the number of researchers dealing with each problem. Table 2 lists the results corresponding to the search terms.

Not surprisingly, most articles discussed the relationship between magnesium and cardiovascular diseases. One reason for this may be that cardiovascular disease is the leading cause of death worldwide. Surprisingly, magnesium and vascular calcification have produced the fewest publications to date. Furthermore, the relationship between magnesium and psychiatric diseases and dementia has been reported in relatively few publications. Cancer is the second leading cause of death worldwide; therefore, the role of Mg in cancer has been highlighted in many publications. The role of magnesium in chronic inflammatory processes has been researched for the longest time, and such articles have been published since the 1920s. However, the role of magnesium in dementia only attracted the attention of researchers a few decades ago, and publications on this topic have only been published since 1967.

## 4. Hypomagnesemia and Diabetes

**Figure 1:**
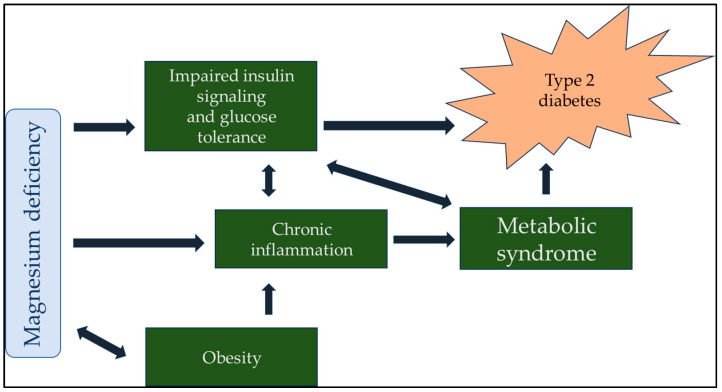
Magnesium plays a central role in the regulation of blood sugar in the body, so it is understandable that a lack of magnesium indirectly contributes to diabetes. Although many factors lead to the development of type 2 diabetes, magnesium can also improve the therapeutic effect of antidiabetic drugs due to its central role.

Maintaining normal insulin sensitivity is impossible without adequate serum and intracellular magnesium levels. Studies in humans and animals have unequivocally demonstrated a robust correlation between low serum magnesium levels and the future development of type 2 diabetes mellitus [16]. Furthermore, a negative correlation was observed between serum magnesium concentration and both fasting blood glucose and postprandial blood sugar values. This finding highlights the potential role of magnesium in the regulation of glucose metabolism and may have implications for the prevention and management of diabetes [17]. Adequate serum magnesium levels can improve insulin sensitivity and decrease blood sugar [18]. Incorporating adequate magnesium supplementation into diabetes management strategies is recommended. Ensuring that individuals with diabetes receive sufficient magnesium supplementation can potentially improve their glucose control and reduce the risk of diabetes-related complications [19]. Moreover, an inverse relationship between glucose–insulin metabolism and magnesium handling should be noted. When blood glucose and insulin levels are high, magnesium is more likely to be excreted through urine. In contrast, magnesium excretion and fasting blood glucose levels have opposite relationships with serum magnesium levels [20]. Maintaining a proper balance of magnesium in the body is crucial for regulating insulin signaling, the phosphorylation of insulin receptor kinase, post-receptor actions of insulin, the cellular uptake of glucose through insulin, and the regulation of glucagon [21]. Sufficient daily intake of magnesium can aid in restoring the optimal concentration of magnesium within cells, resulting in increased insulin-mediated glucose uptake in people with type 2 diabetes. Therefore, high daily magnesium consumption can reduce the risk of developing type 2 diabetes [22]. A meta-analysis of numerous studies showed that the oral consumption of magnesium supplements and the incorporation of the right foods into one’s diet can significantly enhance insulin sensitivity and metabolic control in people with type 2 diabetes [23]. The strong correlation between hypomagnesemia and diabetes is further elaborated upon in Figure 1.

## 5. Hypomagnesemia and Cardiovascular Mortality

The correlation between renal magnesium loss and extensive coronary and vascular calcifications has been firmly established because of the genetic mutation 17q12 [24]. We recently published a narrative review of the connection between hypomagnesemia and cardiovascular disease [25]. In our study, we noted that a lack of magnesium was closely correlated with general atherosclerosis.

There are many correlations between hypomagnesemia and cardiovascular mortality [26]. However, hypomagnesemia causes mitochondrial dysfunction, oxidative stress, and inflammation [27], as discussed in the next section. Several studies have confirmed that hypomagnesemia is an independent risk factor for cardiovascular mortality, including sudden cardiac death [28]. Recent evidence suggests that magnesium plays a clinically significant role in vascular calcification and mortality in individuals with chronic kidney disease (CKD) [29]. Notably, a randomized clinical trial demonstrated the efficacy of oral magnesium oxide in slowing the progression of coronary artery calcification among non-dialysis patients with CKD [30]. This finding implies that magnesium may offer an improved cardiovascular prognosis and thus could prove to be a valuable therapeutic avenue for this patient population. In addition, hemodialysis patients with mild hypermagnesemia exhibited the lowest mortality rate. These findings suggest that magnesium may represent a promising avenue for improving the prognosis of patients with CKD and cardiovascular diseases [31].

If atherosclerosis is caused by diabetes, hyperlipidemia, or chronic kidney disease, hypomagnesemia further aggravates vascular complications. However, the reverse is also true: adequate magnesium supply and supplementation could slow the progression of atherosclerosis. These results confirm that magnesium plays a central role in the development and prevention of vascular calcification.

## 6. Hypomagnesemia and Respiratory Diseases

For almost a century, magnesium administration to asthmatic patients has been shown to effectively decrease the severity of respiratory symptoms. This finding emphasizes the potential of magnesium in the treatment of respiratory diseases [32,33,34]. The use of magnesium alone cannot eliminate severe asthmatic conditions. Therefore, ambivalent results were obtained based on the initial positive results in the early 1990s [35,36]. After much debate, a double-blind, randomized, placebo-controlled trial involving the largest number of patients was conducted to determine the efficacy of magnesium. Although not significantly different from the placebo, the use of magnesium during an acute asthmatic attack can be considered an additional therapeutic option [37]. The significance of magnesium in the treatment of respiratory diseases cannot be ignored, as medical experts highly recommend it as a therapeutic option [38].

It is imperative to note that magnesium has been firmly established as an essential aid in the treatment of respiratory diseases in children [39,40,41,42,43]. However, a positive therapeutic effect is only noticeable when administered intravenously. Compared to an albuterol-based placebo, the hospitalization rate for children with acute refractory asthma was not significantly reduced in 24 h by nebulized magnesium with albuterol in the emergency department [44].

The therapeutic use of magnesium has returned to the spotlight owing to the treatment of patients infected with COVID-19. Magnesium sulfate has the potential to ameliorate several pathophysiological conditions related to the pulmonary vasculature and bronchial smooth muscle. It can facilitate the dilation of constricted pulmonary arteries, reduce resistance within the pulmonary arteries, and induce relaxation in the smooth muscles of the bronchi. Many patients with COVID-19 admitted to hospitals experience pulmonary involvement. COVID-19 can cause hypoxia due to the involvement of the respiratory airways and parenchyma, along with circulatory impairment, causing a ventilation–perfusion mismatch [45]. The severity of COVID-19 infection and prolonged hospitalization are associated with serum magnesium levels [46,47]. According to some authors, population-wide hypomagnesemia could have contributed to the severity of the COVID-19 pandemic [48].

**Figure 2:**
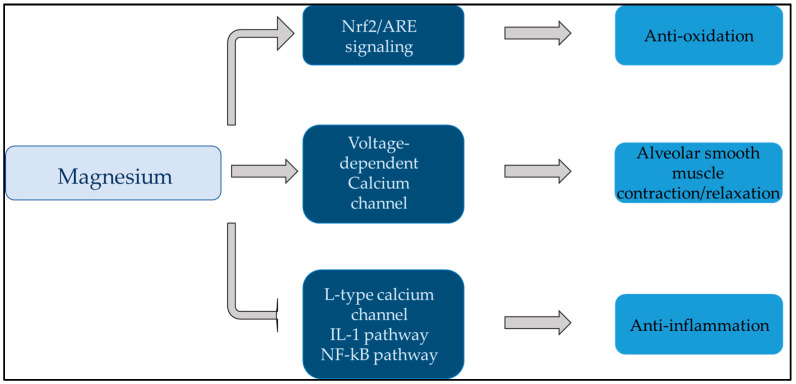
This figure summarizes the effect of magnesium on the lungs. Magnesium plays a central role in anti-inflammation, antioxidation, and alveolar smooth muscle relaxation of the lungs. Abbreviations: ARE: antioxidant response element; IL-1: interleukin-1; Nf-kB: nuclear factor kappa-light-chain-enhancer of activated B cells; Nrf2: nuclear factor E2-related factor 2.

The effects of Mg on the lungs have been discussed in numerous studies. Mg affects bronchial smooth muscle cells by modulating their contractile state. It is widely recognized that magnesium plays a significant role in the physiological functioning of the bronchi. Specifically, magnesium helps to regulate bronchial smooth muscle tone and airway reactivity, which can impact breathing and respiratory health. Research has also shown that magnesium may have anti-inflammatory effects on the airways, further emphasizing its importance in supporting bronchial function [49,50,51]. Figure 2 illustrates the role of Mg in the lungs in a simplified manner.

Consequently, research to date has confirmed a compelling association between magnesium and lung disease. Although low serum magnesium levels are common, they are frequently neglected in critically ill ICU patients. Exposure to a high fraction of oxygen damages the respiratory epithelium and causes long-term fibrosis and mortality in animals and humans. In contrast, high-dose magnesium therapy has been found to alleviate the adverse effects of hyperoxia [52]. Hypomagnesemia has been found to be associated with a higher risk of mortality, sepsis, mechanical ventilation, and extended intensive care unit (ICU) stays for patients who are admitted to the ICU. Failure to do so may result in adverse outcomes, including an increased risk of mortality and prolonged hospital stays [53]. These findings highlight the importance of the early recognition and management of hypomagnesemia in critically ill patients to improve their clinical outcomes and enhance their overall quality of life.

## 7. Hypomagnesemia and the Immune System

Studies conducted on animals have conclusively shown that the relative depletion of magnesium may contribute to inappropriate triggering of the innate immune system while impairing the adaptive immune system, leading to systemic inflammation. Specifically, magnesium deficiency can activate polymorphonuclear leukocytes within the innate immune system, increasing phagocytosis and oxidative stress [54,55]. Magnesium is an essential cofactor in T helper cell adhesion, immunoglobulin synthesis, IgM lymphocyte binding, antibody-dependent cytolysis, and macrophage responses to lymphokines [56,57]. Protein of the M member 7 transient receptor potential cation channel subfamily (TRPM7) is vital for maintaining magnesium levels in immune cells. In the absence of TRPM7, B cell lines experience a decrease in free cytosolic magnesium levels and cell cycle arrest. However, this effect can be reversed by growing cells in high-magnesium medium [58]. Interruption of T-cell development during the CD4 CD8 stage was observed in animal models with a T-cell-specific deletion of the TRPM7 channel. The outcome was a decrease in CD4+ and CD4+ CD8+ cell levels of CD4+ and CD4+ CD8+ cells in the thymus [59]. CD8+ T cells are vital for the adaptive immune system as they play a crucial role in the detection and elimination of cancerous or infected cells. Surveillance of peripheral tissues by effector memory CD8+ T cells is challenging because it requires adaptation to constantly changing microenvironments with highly variable nutrient and oxygen levels [60]. The function of CD8+ T cells can be regulated by extracellular magnesium through a pathway mediated by leukocyte function-associated antigen 1 [61]. The immune system must have sufficient magnesium levels to function properly.

Emerging evidence indicates that the Mg status of an individual is directly associated with its immune function. This implies that Mg may play a crucial role in bolstering immune defense mechanisms. Owing to the global pandemic caused by COVID-19, magnesium has become a subject of renewed interest. In a significant number of patients hospitalized with SARS-CoV-2 infection, hypomagnesemia was present upon admission and tended to deteriorate during hospitalization. Magnesium deficiency within the body can exacerbate the inflammatory response, which may have played a role in the cytokine storm witnessed during severe SARS-CoV-2 illness [62]. Furthermore, magnesium deficiency increases susceptibility to COVID-19 [63]. In the context of immune system modulation, a magnesium concentration exceeding 10 mM and bound to lymphocytes does not effectively buffer the external decrease. This phenomenon has been demonstrated in an animal experiment, wherein the observed result was a reduction in T cell activation and, by extension, a diminished immune response [64].

**Figure 3:**
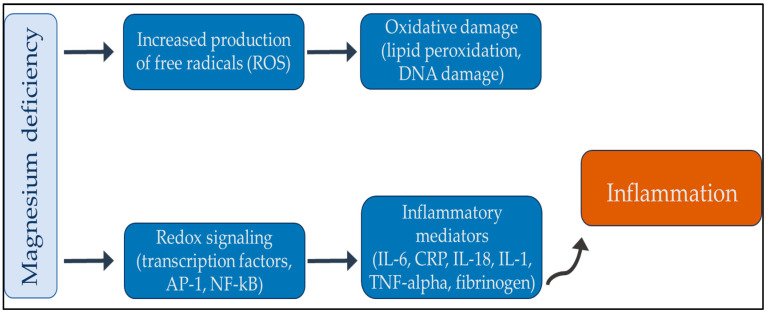
Summary review between magnesium deficiency and inflammation. In summary, an increase in oxidative stress can produce inflammatory mediators, which can cause low-grade chronic inflammation. With an adequate supply of magnesium, the chronic inflammatory process can be interrupted. Abbreviations: AP-1: activator protein 1; CRP: reactive protein; DNA: deoxyribonucleic acid; IL: interleukin; Nf-kB: nuclear factor kappa-light-chain-enhancer of activated B cells; TNF-alpha: tumor necrosis factor-alpha.

Finally, magnesium not only plays a vital role in the normal functioning of the immune system but also triggers the activity of many autoimmune diseases. A growing body of literature has established a strong correlation between magnesium deficiency and rheumatoid arthritis. Multiple articles have been published on this topic, all of which have consistently demonstrated a significant connection between these two entities. Therefore, addressing magnesium deficiency in patients with rheumatoid arthritis may be a useful therapeutic strategy to mitigate symptoms and improve patient outcomes [65,66,67,68]. Moreover, hypomagnesemia is a possible risk factor for severe infections in patients with systemic lupus erythematosus [69]. Finally, a lack of magnesium also contributes to autoimmune thyroiditis, a common disease among young women [70,71,72]. Figure 3 shows the relationship between magnesium and inflammation.

## 8. Hypomagnesemia and Cancer

Concerning the risk of malignancy, it is essential to note that there is a connection between magnesium intake and the development of specific types of cancer. However, it must be understood that the relationship between magnesium and cancer is complex, with multiple uncertainties and qualifiers present that prevent a definitive conclusion. In animal studies, magnesium has been observed to have both positive and negative effects on tumor growth and development. Specifically, while magnesium can impede tumor growth at its original location, it can also facilitate tumor establishment at metastatic sites in various rodent models [73,74,75]. As discussed in the previous section, Mg plays a vital role in the normal functioning of the immune system. General and chronic inflammation have been well established to play a significant role in the growth and spread of cancer. Inflammatory mediators can encourage the invasion of cancer cells into the environment during the early stages of the disease. An absence of magnesium in the body can trigger the activation of tumor necrosis factor (TNF), interleukin-1 (IL-1), and IL-6, which can further enhance the likelihood of cancer cell spread [54,76,77]. Some studies have shown that low magnesium levels can increase the risk of specific types of cancers, such as colorectal, pancreatic, and breast cancers. Therefore, it is vital to maintain adequate Mg levels in the body [78]. With effective magnesium supplementation, the formation and recurrence of colorectal carcinoma can be prevented [79,80,81,82]. Other studies have found that an increase in the incidence of hepatocellular carcinoma can be attributed mainly to the presence of non-alcoholic fatty liver disease (NAFLD). Patients with NAFLD and higher serum magnesium levels have a significantly lower risk of developing cancer [83]. Previous research has also confirmed that increased magnesium intake is associated with a lower incidence of primary liver cancer [84]. In addition, the development of primary lung cancer is significantly related to hypomagnesemia, the background of which can be confirmed by reduced DNA repair capacity [85].

Vitamin C, also known as l-ascorbic acid (AA), possesses antioxidant and pro-oxidant properties. Its effect on cancer cells is based on a hormetic result, meaning that it is stimulated at low doses but inhibited at high doses. Therefore, AA is only effective in high quantities because of its pro-oxidant properties against cancer cells. Magnesium can also enhance the anticancer effects of AA in clinical settings [86]. According to experimental studies, MgCl_2_ triggers apoptosis in cell culture. Additionally, it has been observed to significantly reduce the migration speed of cancer cells with varying metastatic potential [87]. These tests have also shown that magnesium plays a central role in the development of various cancers.

## 9. Hypomagnesemia and Neuropsychiatric Diseases

**Figure 4:**
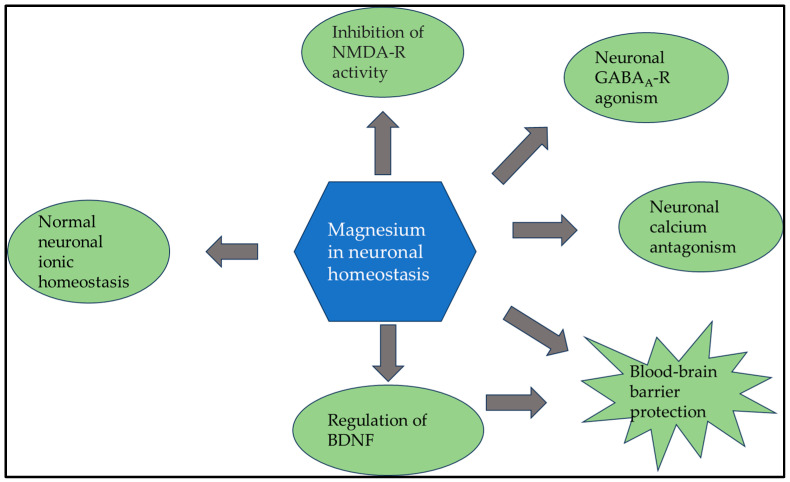
Since magnesium plays a crucial role in normal neuronal functioning at multiple points, it is no surprise that a lack of magnesium can lead to various neurological symptoms. Abbreviations: BDNF: brain-derived neurotrophic factor; GABAA-R: type A receptor; NMDA-R: N-methyl-D-aspartate receptor.

Not surprisingly, magnesium plays a central role in nerve signaling [88,89]. In a complex mechanism at the cellular level, magnesium regulates calcium voltage-dependent channels, while inhibiting the intracellular release of calcium from cytosolic stores. Furthermore, magnesium regulates the most crucial neurotransmitter, the agonist of gamma-aminobutyric acid type A receptor (GABAA-R). In the early developmental process, GABAA-R signaling must be triggered to initiate the release of magnesium from the mitochondria [90]. The augmentation of cytoplasmic magnesium levels triggers the activation of the mechanistic target of rapamycin (mTOR), which subsequently promotes the formation of neural networks via ribosome biogenesis. Simultaneously, magnesium plays a vital role in inhibiting the glutamate N-methyl-D-aspartate receptor (NMDAR), which is responsible for neuronal death. Therefore, it is important to understand the significance of Mg in these processes [91,92]. Furthermore, magnesium is not only of prime importance in neuronal signaling and the viability of neurons, but also plays a vital role in the maintenance and regulation of the blood–brain barrier and neurotrophins, such as brain-derived neurotrophic factor (BDNF), which contributes to neuronal plasticity and plays a central role in learning and memory [93]. The complex role of magnesium in normal neuronal functioning is illustrated in Figure 4.

Hypomagnesemia is associated with the severity and presence of several psychiatric disorders. These symptoms may include insomnia, irritability, anxiety, depression, panic attacks, psychotic behavior, hyperexcitability, headaches, dizziness, and tremors. Neuromuscular peripheral symptoms of hypomagnesemia are well known and established, including muscular weakness, myalgia, and asthenia [94,95]. In animal models, a lack of magnesium can lead to hyperexcitability of the central nervous system (CNS), as indicated by electrophysiological evidence. The electroencephalogram readings showed increased activity, suggesting that magnesium-deficient rats experienced behavioral changes in response to auditory stimulation. These changes may be caused by an increase in CNS excitability related to magnesium deficiency [96]. Research has also linked magnesium deficiency to neuromuscular hyperexcitability in humans [97,98]. Magnesium appears to be effective for the complete treatment of depression and insomnia [99,100,101].

It has been more than 30 years since dementia and hypomagnesemia were linked together [102,103,104]. Changes in magnesium metabolism have been observed in individuals with dementia. Consistently, there have been reduced levels of total and ionized serum magnesium and a decrease in magnesium content in various tissues among patients with Alzheimer’s [105,106]. An experimental study demonstrated the efficiency of magnesium-L-threonate in protecting hippocampal neuronal cell apoptosis against oxidative stress [107]. Studies have shown that magnesium-L-threonate can reduce neuroinflammation and decrease beta-amyloid deposition in animal models of Alzheimer’s disease. In addition, it has been found to improve learning abilities and short- and long-term memory [108,109,110]. Extensive research on the impact of Mg on cognitive health in humans is still lacking. Among dialysis patients, a U-shaped relationship between mild cognitive impairment and serum magnesium levels has been observed, partly reflecting the special features of these patients [111]. Although few clinical trials have been conducted in this area, some studies have suggested that people who consume high-magnesium diets may have a lower risk of experiencing cognitive decline [112,113]. Finally, it appears that magnesium deficiency also plays a role in severe neurological symptoms caused by SARS-CoV-2 [114].

## 10. Summary

As discussed above, without claiming to be complete, the pivotal role of Mg in human health can be appreciated through numerous publications on the subject. In our narrative review, we wanted to highlight the key points of this relationship and the contradiction that magnesium supplementation, a simple and inexpensive maneuver, is not included in daily practice. According to some authors, at least 30% of the adult population does not consume the daily magnesium requirement through their diet [8]. It is vital to note that insufficient magnesium intake can lead to a range of health problems, including muscle cramps, fatigue, and even cardiovascular disease. We also have to add to this the decrease in magnesium that occurs as a side effect of the medications. Proton pump inhibitors, which are most commonly prescribed, are known to cause hypomagnesemia [115,116]. Therefore, every physician should consider an adequate supply of magnesium for their patients and minimize the use of interfering medications, such as proton pump inhibitors, wherever feasible. The optimal magnesium intake should be determined in accordance with the individual’s body weight, typically ranging from 4 to 6 mg per kg/day. Adherence to these guidelines is recommended to ensure adequate magnesium levels and mitigate the risk of magnesium deficiency [4].

## 11. Conclusions

Based on the available data and reports, we conclude that the development of many disease processes can be prevented or slowed by replacing magnesium, an inexpensive supplement. It cannot be emphasized that prevention is more important and cost-effective than treating established diseases. In accordance with the dietary requirements discussed, it is recommended that an average daily intake of 500 mg of magnesium be followed. This recommendation considers the specific nutritional requirements that are necessary for optimal health and well-being. Adherence to this recommendation will ensure that the body receives adequate levels of magnesium, an essential cation with a critical role in numerous physiological processes and the maintenance of human health. 

## Figures and Tables

**Table 1:** Summary of the main factors involved in maintaining the body’s magnesium homeostasis. The most common causes of hypomagnesemia are insufficient daily intake and increased excretion.

- The diet requirements of healthy individuals are 5 to 7 mg/kg of body weight/day magnesium intake. - Gastrointestinal absorption or loss. - Renal excretion or loss. - Hypomagnesemia can be caused by a decrease in tubular reabsorption, osmotic diuresis, or the use of certain medications that lead to magnesium wasting. - Bone is the primary storage site of magnesium. - Extracellular magnesium is in equilibrium with magnesium in storage.

**Table 2:** Search results for the keywords ‘magnesium’, ‘diabetes’, ‘cardiovascular disease’, ‘respiratory disease’, ‘immune system’, ‘inflammation’, ‘autoimmune disease’, ‘neurology’, ‘psychiatry’, ‘cognitive function’, ‘cancer’, and ‘vascular calcification’.

Searched Keywords	Number of Publications until 1 August 2023	First Publication in This Field
Magnesium and diabetes	2880	1947
Magnesium and cardiovascular disease	8400	1946
Magnesium and respiratory disease	2052	1948
Magnesium and the immune system	2974	1951
Magnesium and inflammation	1655	1920
Magnesium and autoimmune disease	597	1956
Magnesium and neurology	1054	1955
Magnesium and psychiatry	492	1950
Magnesium and cognitive function	270	1967
Magnesium and cancer	5080	1934
Magnesium and vascular calcification	206	1956

## Data Availability

Not applicable.
